# Age-dependent remodeling of gut microbiome and host serum metabolome in mice

**DOI:** 10.18632/aging.202525

**Published:** 2021-02-17

**Authors:** Chia-Shan Wu, Sai Deepak Venkata Muthyala, Cory Klemashevich, Arinzechukwu Uchenna Ufondu, Rani Menon, Zheng Chen, Sridevi Devaraj, Arul Jayaraman, Yuxiang Sun

**Affiliations:** 1 Department of Nutrition, Texas A&M University, College Station, TX 77843, USA; 2 USDA/ARS Children’s Nutrition Research Center, Department of Pediatrics, Baylor College of Medicine, Houston, TX 77030, USA; 3 Integrated Metabolomics Analysis Core, Texas A&M University, College Station, TX 77843, USA; 4 Artie McFerrin Department of Chemical Engineering, Texas A&M University, College Station, TX 77843, USA; 5 Department of Biochemistry and Molecular Biology, The University of Texas Health Science Center at Houston, Houston, TX 77030, USA; 6 Department of Pathology, Texas Children’s Hospital, Houston, TX 77030, USA; 7 Department of Pathology and Immunology, Baylor College of Medicine, Houston, TX 77030, USA

**Keywords:** gut microbiome, serum metabolome, aging, metabolism

## Abstract

The interplay between microbiota and host metabolism plays an important role in health. Here, we examined the relationship between age, gut microbiome and host serum metabolites in male C57BL/6J mice. Fecal microbiome analysis of 3, 6, 18, and 28 months (M) old mice showed that the *Firmicutes*/*Bacteroidetes* ratio was highest in the 6M group; the decrease of *Firmicutes* in the older age groups suggests a reduced capacity of gut microflora to harvest energy from food. We found age-dependent increase in *Proteobacteria*, which may lead to altered mucus structure more susceptible to bacteria penetration and ultimately increased intestinal inflammation. Metabolomic profiling of polar serum metabolites at fed state in 3, 12, 18 and 28M mice revealed age-associated changes in metabolic cascades involved in tryptophan, purine, amino acids, and nicotinamide metabolism. Correlation analyses showed that nicotinamide decreased with age, while allantoin and guanosine, metabolites in purine metabolism, increased with age. Notably, tryptophan and its microbially derived compounds indole and indole-3-lactic acid significantly decreased with age, while kynurenine increased with age. Together, these results suggest a significant interplay between bacterial and host metabolism, and gut dysbiosis and altered microbial metabolism contribute to aging.

## INTRODUCTION

Aging is a complex trait that is influenced by individual genetics, diet, lifestyle, and environmental factors. The increase in life expectancy is often accompanied with additional years of susceptibility to chronic disorders such as obesity, insulin resistance, and cognitive impairment [1–4]. Furthermore, aging is increasingly recognized as being associated with a pro-inflammatory state that promotes the development of chronic diseases, hence the term “inflammaging” [5]. During the past decade, accumulating evidence suggests that the gut microbiota impacts body weight, energy homeostasis, innate immune system and aging [6–8]. For example, epidemiological studies show that loss of diversity in the core microbiota groups is associated with increased frailty [9, 10]. It is important to note that gut microbiota do not age per se, but people growing older may experience comorbidities associated with the gut and with gut bacteria [11]. How the microbiota in the gut affect the aging process, or whether gut microbiota simply change as a function of age, remain unclear.

Recently, advances in analytical platforms have accelerated metabolomics studies, which aim to systematically identify and quantify a global set of metabolites or low-molecular-weight intermediates (molecular weight < 1.5 kDa) in a given biological system in response to pathophysiological stimuli, genetic modification, or environmental factors [12]. Several metabolomics studies reported age-related changes in metabolite levels and found strong correlation between metabolomics profiles and chronological age as well as longevity [13–18]. Signature metabolites that are associated with aging include amino acids, lipids, carbohydrate, TCA cycle, and redox metabolism; however, how these diverse metabolites regulate the aging process and its complex networks remains unclear. Furthermore, most of the serum metabolome studies assess the fasting state, using blood samples collected after overnight fasting [13–17]. Interestingly, a previous study comparing plasma metabolomes of conventional and germ-free mice at the fed state demonstrates a significant effect of the gut microbiome on mammalian blood metabolites [19]. For example, the bacterial-mediated production of bioactive indole-containing metabolites derived from tryptophan is significantly impacted, and colonization of bacterium *Clostridium sporogenes* restores the production of indole-metabolites. Hence, these results suggest a significant interplay between bacterial and host metabolism, when plasma profile was assessed at the fed state.

In this study using mice at different ages, we first defined the core microbiota changes associated with aging, followed by serum metabolome profiling at the fed state. We found age-dependent changes in microbial taxa associated with gut dysbiosis and pro-inflammatory signaling, and age-associated decrease in circulating levels of the gut microbe-dependent metabolites indole and indole-3-lactic acid. Signature pathways associated with aging include tryptophan metabolism, purine metabolism, amino acids, and nicotinate and nicotinamide metabolism. Together, these data suggest that gut dysbiosis and altered gut microbial metabolism may contribute to the metabolic dysfunction and inflammation in aging.

## RESULTS

### Age-associated changes in microbial biodiversity and composition

**Figure 1:**
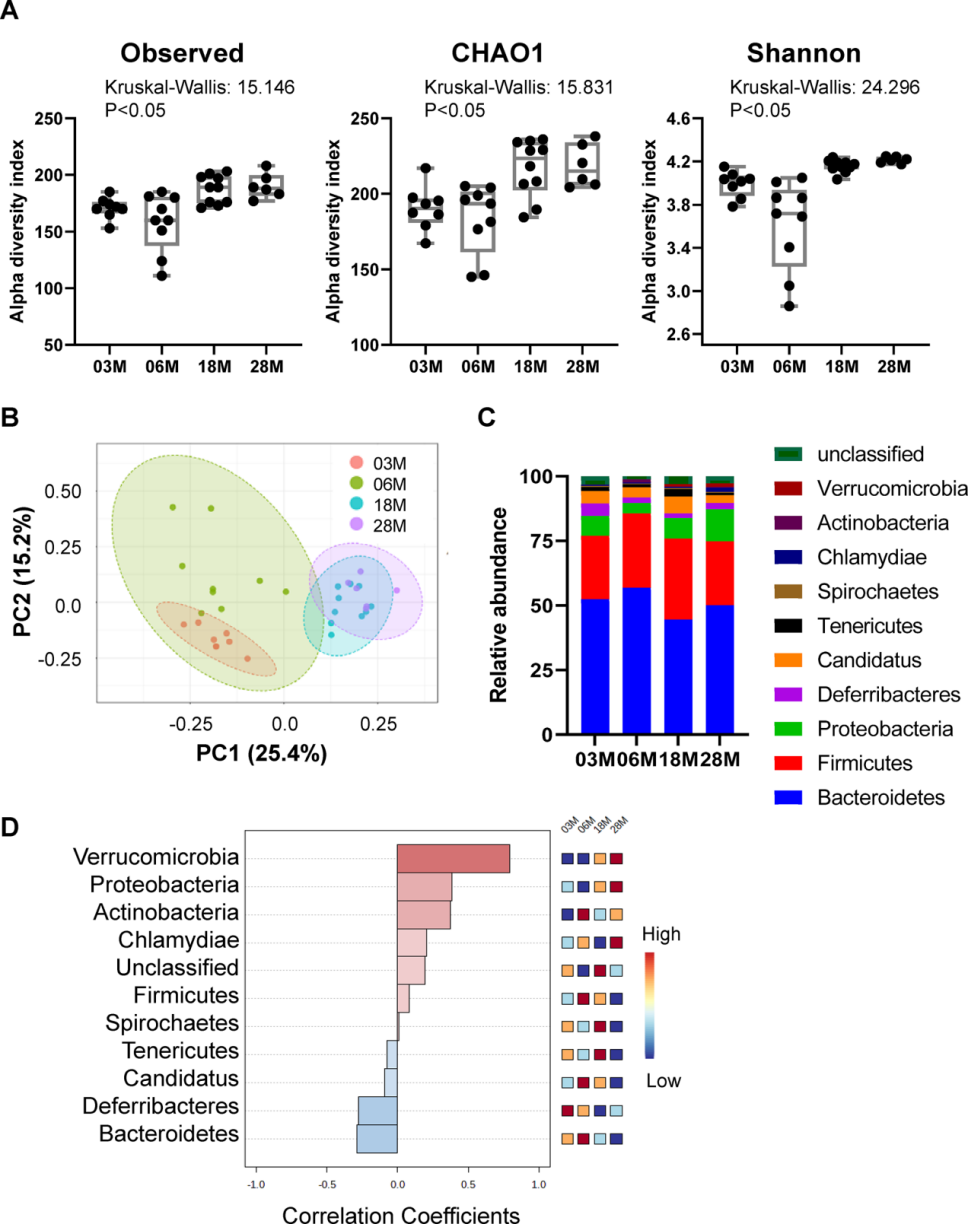
**Alteration in gut microbiota diversity and composition at different ages.** Mice at 3-, 6-, 18- and 28 months of age (3M, 6M, 18M, 28M) were used (n=8, 9, 10 and 6, respectively). (**A**) Box-and-whisker plot showing the bacterial α-diversity measurements, including richness (observed species and chao1), and overall sample diversity measured according to Shannon metrics. One-way ANOVA with age as an independent factor. (**B**) PCoA analysis plot representing microbial β-diversity. (**C**) Microbiome composition at the level of major phyla. (**D**) Correlation analysis of the abundance of the major phyla with age. Heatmap shows the abundance-fold-change of bacteria with age. In red: bacteria which are more abundant, in blue: bacteria which are less abundant. Scale: Log2 (Fold change) = -1 (blue) < 0 (white) < 1 (red).

To examine the impact of age on mouse gut microbiota, the variable region 4 (V4) of bacterial 16S rRNA genes in fecal samples from 3M, 6M, 18M and 28M C57BL/6J male mice were amplified by PCR and sequenced using the Bio Scientific NEXTFlex^TM^ platform. These mice were born and raised in house, and fecal samples were collected on the same day and stored at -80° C until processing. To investigate how aging affects the microbiota phylogenetic richness and diversity in each fecal sample, we analyzed the α-diversity (Figure 1A). The Observed index is a richness-based measure which calculates the actual number of unique taxa observed in each sample, while Chao1 analysis predicts the number of taxa in a sample by extrapolating the number of rare organisms that may have been missed due to under sampling [20]. Shannon diversity index is a hybrid measure of the richness of a sample and the evenness of taxa in the sample. Interestingly, the evenness index of Shannon and the richness measures of Observed index and Chao1 index all showed significant age-dependent increases from 3M to 28M.

To compare the group differences in bacterial communities, the β-diversity of microbial composition was calculated by using principal coordinate analysis (PCoA). A clear age-dependent separation between the communities was observed at the first principal coordinate (PC1 axis), which explained 25.4% of the variance (Figure 1B). Moreover, there is an age-dependent shift in the 4 age groups, from left to right, on the PC1 axis; the 18M and 28M groups overlapped on the PC1 axis. Interestingly, 6-months old mice showed a greater individual variance as suggested by the larger green eclipse in Figure 1B, and larger box-whisker in Figure 1A. Analysis of microbial composition at the phylum level indicated that the fecal microbiota was dominated by 5 major phyla: *Bacteroidetes*, *Firmicutes*, *Proteobacteria*, *candidatus Saccharibacteria* and *Deferribacteres* (Figure 1C). Correlation analysis showed that there were age-associated increases in *Verrucomicrobia* and *Proteobacteria*, and age-associated decreases in *Bacteroidetes* and *Deferribacteres* (Figure 1D). Interestingly, the abundance of *Firmicutes* and *Candidatus* peaked at 6M, then age-dependently decreased in 18M and 28M groups.

**Figure 2:**
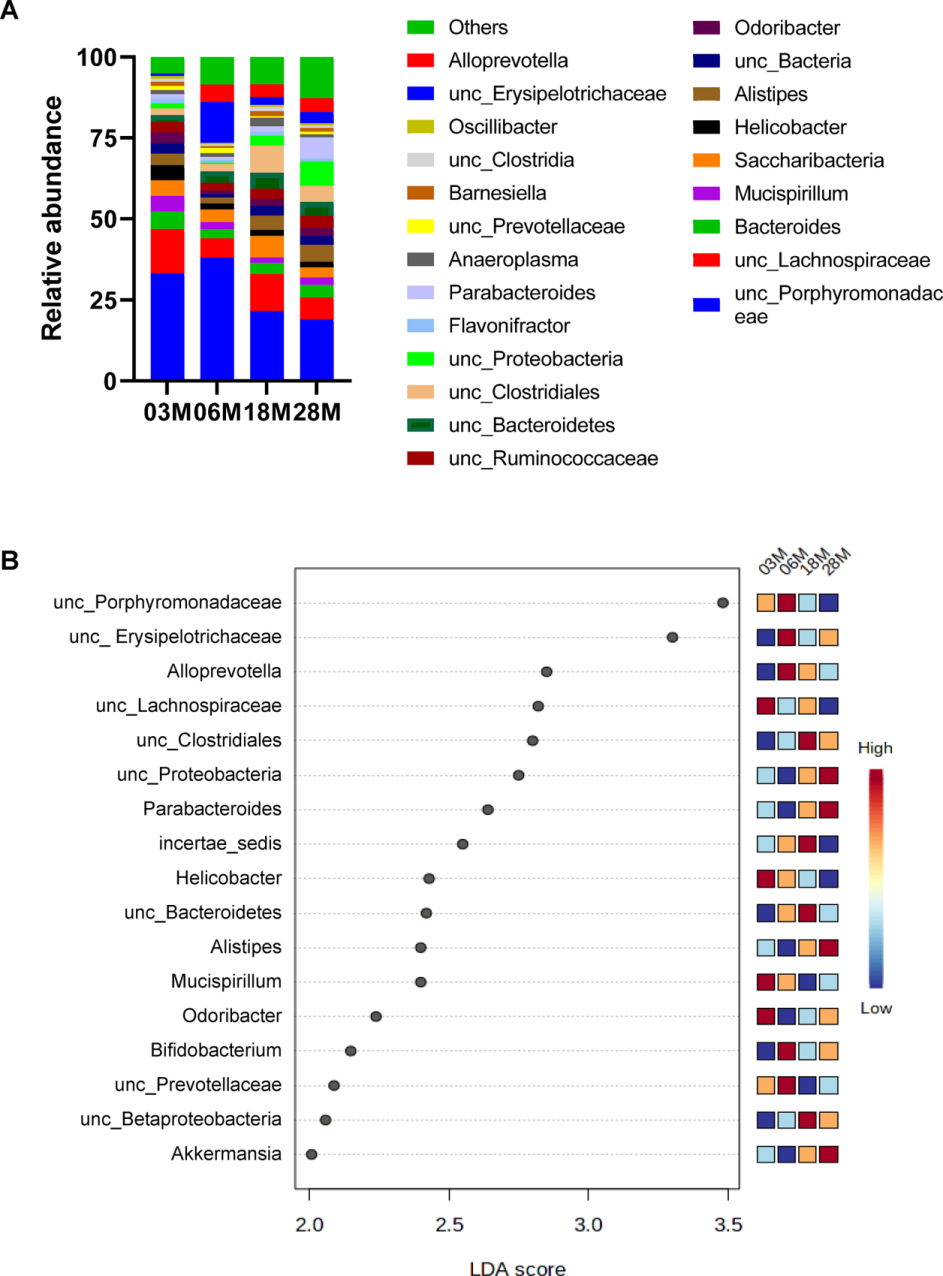
**Taxonomic distribution of fecal microbiome by genera.** (**A**) Microbiome composition at the level of genera. (**B**) The left histogram shows the Linear discriminant analysis (LDA) effect size (Lefse) scores computed for features (on the OTU level) differentially abundant between the different age groups. The right heatmap shows the relative abundance (log10 transformation) of OTUs. “unc”: unclassified.

Next, we compared microbial differences at the genus level (Figure 2A). To identify specific bacterial taxa or genus-level phylotype that contributes to age, we applied Linear discriminant analysis Effect Size (LEfSE); 17 significant genera were identified as shown in Figure 2B. There were age-dependent increases in *Proteobacteria*, *Parabacteroides*, *Allistipes* and *Akkermansia*. On the other hand, there were age-dependent decreases in *Porphyromonadaceae*, *Mucispirillum*, and *Prevotellaceae*.

### Age-associated changes in serum metabolome

To identify metabolites whose abundances may be associated with aging, we profiled serum metabolites at the fed state from 3M, 12M, 18M and 28M male mice, using untargeted LC-MS metabolomics. We obtained abundance measurements for 9482 metabolite features, 1820 of which were annotated with Compound Discoverer (Supplementary Table 1).

**Figure 3:**
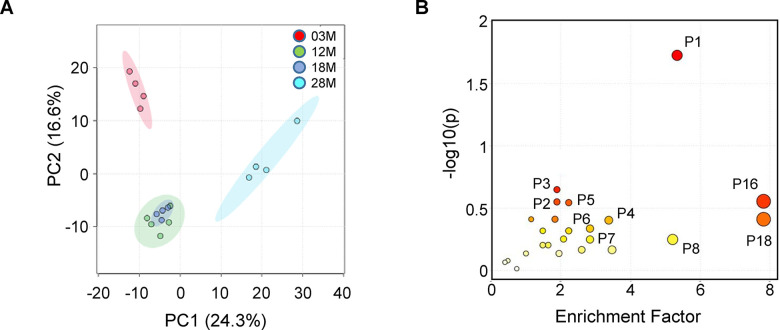
**Fed-state serum metabolome and enriched pathways impacted by aging.** Serum samples from 3-, 12-, 18- and 28 months old (3M, 12M, 18M and 28M) mice at the fed state were analyzed (n=4, 5, 4 and 4, respectively). (**A**) PCA analysis shows the grouped discriminations of the different age groups. 12M and 18M groups clustered together on the PC1 axis. (**B**) Mummichog pathway analysis plot, using peaks-to-pathway analysis module in MetaboAnalyst 4.0. The color and size of each circle corresponds to its p-value and enrichment factor, respectively. The enrichment factor of a pathway is calculated as the ratio between the number of significant pathway hits and the expected number of compound hits within the pathway [69]. The corresponding pathways are listed in Table 1.

We first analyzed the data using Principal Component Analysis (PCA), which is an unsupervised method to interpret the variance in a dataset (X) without referring to class labels (Y). PCA showed that 12 and 18M age groups clustered together, while there was clear difference between the 3M, 12-18M and 28M age groups in the first two principal components, PC1 (24.3%) and PC2 (16.6%). This suggested that there were age-associated changes in metabolites in serum (Figure 3A). Next, for each of these metabolites, we computed the mean fold change (in log 10 space) and the statistical significance between the 4 age groups using one-way ANOVA followed by Tukey’s post-hoc tests. This resulted in 510 metabolites with a log(p-value) above 1.8 (FDR-adjusted *p* value < 0.05, Supplementary Table 2). To explore the potential pathways disturbed in aging, the metabolites and *p*-values were analyzed using MetaboAnalyst 4.0 to identify differentially altered pathways. The MS Peaks to Pathways analysis predicts biological activity directly from peak list data, thereby bypassing metabolite identification. This analysis identified 6 pathways that are impacted by aging (Gamma values < 0.05, Figure 3B and Table 1). The top ranked pathways include tryptophan metabolism, purine metabolism, and amino acids metabolism (Table 1).

**Table 1:** Results of the mummichog pathway analysis.

**Pathway**	**Pathway total**	**Hits total**	**Hits sig**	**Expected**	**FET**	**EASE**	**Gamma**	**Pathway Number**	**Compound Hits**
Tryptophan metabolism	41	20	7	1.309	0.019	0.057	0.002	P1	C00078;C05635;C03824;C04409;C00328;C05653;C05647;C00108
Purine metabolism	66	18	4	2.108	0.284	0.516	0.010	P2	C00294;C00387;C00147;C02350
Cysteine and methionine metabolism	33	6	2	1.054	0.227	0.622	0.016	P3	C01137;C00073
Arginine and proline metabolism	37	21	4	1.182	0.398	0.630	0.016	P4	C01137;C05147;C01157;C03440;C03912;C01165;C00884
Alanine, aspartate and glutamate metabolism	28	7	2	0.894	0.288	0.679	0.020	P5	C01042;C03912
Aminoacyl-tRNA biosynthesis	22	10	2	0.702	0.465	0.805	0.038	P6	C00073;C00078
Pentose phosphate pathway	22	12	2	0.702	0.569	0.860	0.052	P7	C00345;C00279
Caffeine metabolism	12	12	2	0.383	0.569	0.860	0.052	P8	C16361;C07481
Steroid hormone biosynthesis	77	12	1	2.459	0.868	1.000	1.000	P9	C11133
Arginine biosynthesis	14	4	1	0.447	0.485	1.000	1.000	P10	C00437
Glycine, serine and threonine metabolism	31	8	1	0.990	0.737	1.000	1.000	P11	C00430
Lysine degradation	19	6	1	0.607	0.631	1.000	1.000	P12	C03366
Histidine metabolism	16	8	1	0.511	0.737	1.000	1.000	P13	C01262
Tyrosine metabolism	42	22	1	1.341	0.977	1.000	1.000	P14	C00355
Phenylalanine metabolism	12	7	1	0.383	0.689	1.000	1.000	P15	C00166;C02763
Phenylalanine, tyrosine and tryptophan biosynthesis	4	2	1	0.128	0.281	1.000	1.000	P16	C00166
beta-Alanine metabolism	21	6	1	0.671	0.631	1.000	1.000	P17	C01262
D-Arginine and D-ornithine metabolism	4	3	1	0.128	0.391	1.000	1.000	P18	C01110
Vitamin B6 metabolism	9	7	1	0.287	0.689	1.000	1.000	P19	C00847
Nicotinate and nicotinamide metabolism	15	5	1	0.479	0.564	1.000	1.000	P20	C00153

List of pathways that are enriched in the aging metabolome dataset, ranked by the gamma-adjusted p-values. The table includes the total number of hits per pathway (all, significant, and expected), the raw p-values (FET), and the gamma-adjusted p-value (for permutations) per pathway.

**Figure 4:**
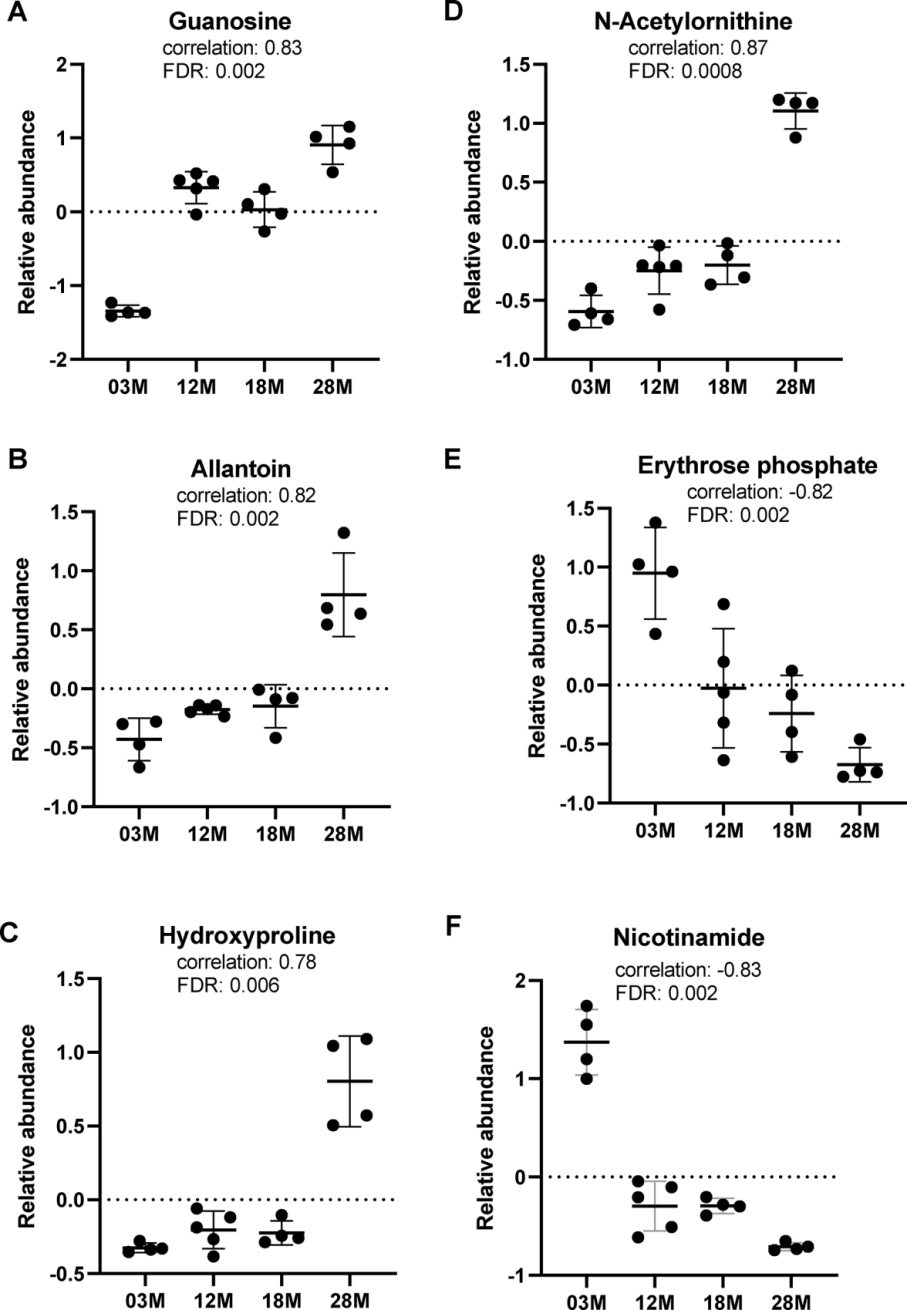
**Top metabolic alterations in aging.** (**A**, **B**) Guanosine and allantoin, metabolites in the purine metabolism pathway. (**C**, **D**) Hydroxyproline and N-acetylornithine, metabolites in the arginine and proline metabolism pathway. (**E**, **F**) Erythrose phosphate and nicotinamide, metabolites in the pentose phosphate and nicotinamide pathways, respectively. n=4, 5, 4 and 4 for the 3-, 12-, 18- and 28 months old (3M, 12M, 18M, 28M) groups, respectively.

We further assessed the data for correlations of metabolites to age, using the pattern-hunter algorithm in MetaboAnalyst. This pattern search identified 372 significant features with positive or negative correlation coefficients between 1 and 0.6. We extracted the relative abundance of metabolites in tryptophan, purine, arginine and proline metabolism identified in the pathway analysis to show the direction of change. Of note, tryptophan and indole were positively identified by matching the spectra from pure standards and chromatographic retention time, while the other metabolites are putatively annotated by matching to either the spectral library (mzCloud) or accurate mass (ChemSpider). We found that guanosine and allantoin, two metabolites in the purine metabolism, showed significant age-associated increase in abundance (Figure 4A, 4B). Hydroxyproline and N-acetylornithine, metabolites in arginine and prolife metabolism, also showed age-associated increase in abundance (Figure 4C, 4D). Erythrose phosphate, metabolite in the pentose phosphate pathway (P7 in Table 1), showed significant age-associated decrease in abundance (Figure 4E). On the other hand, nicotinamide, a metabolite in the nicotinamide pathway (P20 in Table 1) and a significant feature identified by the correlation pattern hunter algorithm, showed significant age-associated decrease in abundance (Figure 4F).

**Figure 5:**
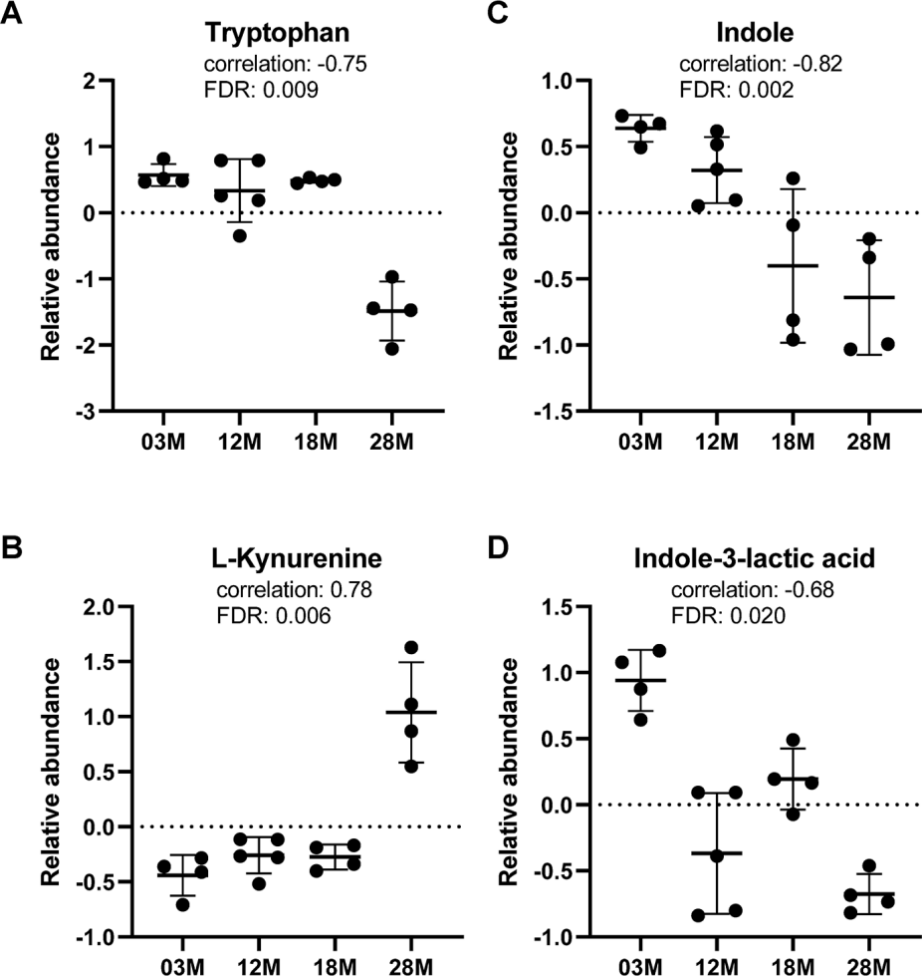
**Tryptophan metabolism was altered in aging.** (**A**) Tryptophan and (**B**) L-kynurenine, metabolite from tryptophan catabolism by host. (**C**, **D**) indole and indole-3-lactic acid, metabolites from tryptophan catabolism by gut bacteria. n=4, 5, 4 and 4 for the 3-, 12-, 18- and 28 months old (3M, 12M, 18M, 28M) groups, respectively.

Regarding tryptophan metabolism, we found age-associated decrease in serum levels of tryptophan, while L-kynurenine, a metabolite in the tryptophan catabolism pathway, showed age-associated increase in abundance (Figure 5A, 5B). Notably, indole and indole-3-lactic acid, metabolites in the bacterial tryptophan catabolism pathway, showed significant age-associated decreases in serum abundance levels (Figure 5C, 5D).

## DISCUSSION

Here we explored, under controlled environmental conditions, changes in mouse gut microbiome and serum metabolome with age. We found that aging was associated with increased alpha diversity, a measure of the richness of species present. This is consistent with previous studies reporting greater alpha diversity with age in mouse gut microbiome [21–24]. On the other hand, human microbiome has higher complexity, due to the myriad of external factors influencing microbial populations between aging individuals [25, 26]. Jeffery et. al [10] applied an iterative bi-clustering algorithm to microbiota composition data from the ELDERMET cohort to identify 4 subpopulations within the microbiota that are associated with biological phenotypes; moreover, loss of diversity in the core microbiota groups is associated with increased frailty, but not significantly associated with chronological aging [11]. Notably, the *Clostridiales* subpopulation is significantly associated with increased frailty, to a much greater extent than the loss of gut microbiota diversity. Our data showed age-associated increase in *Clostridiales* from 3 to 18M, then decreased in the 28M group. A previous study has shown that centenarians have a microbiota that differs from those of older adults [9]. Our data also showed that *Alloprevotella* peaked at 6M, then age-dependently decreased in the 18M and 28M group. Interestingly, *Alloprevotella* has been associated with a decreased lifetime cardiovascular disease risk in the participants of the Bogalusa Heart Study [27].

At the phylum level, we observed that the *Firmicutes*/*Bacteroidetes* (F/B) ratio is highest in the 6M group, and age-dependently decreased in the 18M and 28M groups. Previous study showed that the F/B ratio evolves during different life stages: the ratios were 0.4, 10.9 and 0.6 for infants, adults and elderly individuals, respectively [28]. Increased F/B ratio has been associated with excess energy harvest from food in obese patients [29, 30]. The phylum *Firmicutes* includes *Clostridium* cluster XIVa, which contains many butyrate producing species [30]. We speculate that the decrease in *Firmicutes* in the 28M group may reflect a decreased capacity of the gut microflora at the extreme old age to harvest energy from food.

The age-dependent increase in *Proteobacteria* is consistent with a previous study showing significantly higher levels of *Proteobacteria* in the centenarian population [9]. Interestingly, *Proteobacteria* have been associated with altered mucus structure that is more penetrable by bacteria, leading to increased intestinal inflammation [31]. Furthermore, transferring aged microbiota to young germ-free (GF) mice lead to inflammation in the small intestine in the GF mice, increased leakage of inflammatory bacterial components into the circulation, and promoted increased T cell activation in the systemic compartment; these effects are associated with higher abundance of *Proteobacteria* in the aged microbiota after transfer [32].

In contrast to previous reports showing decreased abundance of *Akkermansia* in aging [23], here we found age-associated increase. *Akkermansia* are typically linked to health beneficial and anti-inflammatory effects [33], such as improving glucose homeostasis in diet-induced obesity [34], and slowing progression of dextran sulfate sodium-induced colitis [35], although the mucin degradation properties of the members of this genus may also exacerbate certain types of infection [36]. Future studies are needed to further explore these differing findings. Taken together, our results indicate that older mice demonstrated several bacterial markers of gut dysbiosis and inflammation.

We found age-dependent decrease in serum nicotinamide, consistent with previous studies showing age-related decline in nicotinamide adenine dinucleotide (NAD^+^) levels [37]. Emerging evidence suggests that NAD^+^ is a critical signaling molecule and essential substrate for sirtuins, a class of enzymes that mediate several of the beneficial effects of calorie restriction in model organisms, including the maintenance of cardiovascular function [38]. Thus, the age-related decline in the cellular bioavailability of NAD^+^ and related metabolites in animals and in humans may contribute to physiological aging by reducing sirtuin activity [37, 39–41]. NAD^+^ can be synthesized de novo from the amino acid tryptophan, or from a salvage pathway for regenerating NAD^+^ from other intracellular intermediates, which are primarily made available through dietary sources [42]. Importantly, supplementation of nicotinamide intermediates has been shown to improve cardiac health in older humans [43] and in aged mice [44]. Furthermore, a study of 12 adult men, ranging from 80-90 years of age, showed an anti-inflammatory effect of nicotinamide riboside supplement [45].

One of the significant pathways associated with aging identified in our metabolome data is purine metabolism. We found significant increases in allantoin and guanosine levels in older mice. Purines are endogenous organic molecules that are essential for all cells; they are structural constituents of nucleic acids, and acts as second messengers in intracellular signaling pathways [46]. Purines consist of the two-ring nitrogenous bases adenine, guanine, and their derivatives such as adenosine and guanosine. Interestingly, in a study profiling the metabolome of mouse brain at different stages of the life cycle (12, 18 and 24 months) and across different anatomical regions (hippocampus, frontal cortex and caudoputamen) revealed metabolic imbalance in the aging brain, characterized by NAD^+^ decline, increased AMP/ATP, and purine/pyrimidine accumulation [47]. Thus, while the physiological significance of elevated levels of allantoin and guanosine in circulation is currently not clear, our metabolome data may reflect a metabolic imbalance in the aging mice.

Pathway analysis of our metabolome data showed tryptophan metabolism to be a major pathway altered in aging. Correlation analyses revealed that, while there is age-associated increase in kynurenine levels, there are significant age-associated decreases in tryptophan, indole and indole-3-lactic acid levels. The regulation of tryptophan concentration is critical for the maintenance of systemic homeostasis, since it is involved in several pathways including nutrient sensing, metabolic stress response, and immunity [48, 49]. The majority (>95%) of dietary tryptophan is fed into the host kynurenine pathway, giving rise to a number of downstream metabolites, while approximately 4–6% of tryptophan is available to the microbiota for biotransformation [48, 50, 51]. In addition to tryptophan being important as a precursor for the synthesis of the neurotransmitter serotonin, several catabolites along the kynurenine axis are neuroactive [52]. Furthermore, aging affects tryptophan metabolism, giving rise to higher kynurenine and lower tryptophan concentrations that are associated with neuropsychiatric symptoms [52]. On the other hand, indole is produced by a variety of both gram-positive and gram-negative bacteria with tryptophanase activity, a bacteria-specific enzyme that catabolizes tryptophan to indole, pyruvate, and ammonia [50, 51]. Indoles have been reported to regulate virulence in pathogenic bacteria [53, 54], protect hosts from infection, and limit colitis induced by pathogens or chemical stressors [55–57]. The effects of indole and metabolites are thought to proceed largely via the aryl hydrocarbon receptor, and IL-22 signaling [58, 59]. Interestingly, indoles have been shown to extend healthspan in worms, flies and mice via the aryl hydrocarbon receptor, without significantly altering lifespan [60]. In addition, indole enhances barrier functions of gut epithelium by inducing the expression of genes involved in tight junction, adherens junctions, actin cytoskeleton and mucin production [57, 61]. Taken together, our metabolome data showing age-associated decrease in indole and its metabolite levels may reflect changes in gut microbial metabolism, and compromised gut barrier function in aging.

Pathway analysis showed several amino acid metabolism pathways to be significantly impacted by aging; however, correlation analyses identified limited metabolites in these pathways. Interestingly, we found age-associated increases in hydroxyproline. Hydroxyproline is one of the most abundant amino acids found in collagen, and increased levels of hydroxyproline in blood has been associated with connective tissue degradation [62]. At cellular level, hydroxyproline may also scavenge oxidants and regulate the redox state of cells [63]. Hence, metabolome analyses at tissue levels are required to complement and confirm the findings from serum metabolome. Furthermore, our serum metabolome data, at fed state, reflects systemic metabolites from both host and microbiome, and causal links between gut microbiome and serum metabolome cannot be inferred from our current data. Future interventional studies, such as fecal transplantation from young or old mice into microbiota-depleted old or young mice, are required to decipher the causal relationship between gut microbiome and metabolome changes in aging. For future follow-up study, we plan to transplant fecal pellets collected from 18 months-old donor mice, into 3 months-old recipient mice that have been treated with a cocktail of broad spectrum antibiotics as described [64], and assess fecal microbiome and metabolome before and after fecal transplantation.

In summary, here we showed that gut microbiome composition is altered by primary aging in mice, including changes in microbial taxa associated with gut dysbiosis and pro-inflammatory signaling. We showed that aging is associated with a decrease in circulating levels of the gut microbiota-dependent metabolites indole and indole-3-lactic acid, which may reflect compromised gut barrier function and pro-inflammatory state. Collectively, these data suggest that gut dysbiosis and altered gut microbial metabolism may contribute to the metabolic imbalance in the aging mice. Our findings support the development of gut microbiome-targeted agents and interventions to promote healthy aging.

## MATERIALS AND METHODS

### Animal

Male C57BL/6J mice at different ages were used in the study. Mice belonging to the same age group were housed together (3-5 mice/cage). Animals were housed under controlled temperature and lighting (75 ± 1° F; 12-hour light–dark cycle, lights on at 6:00 AM) with free access to food and water. All diets were from Harlan Teklad (2920X, 16% of calories from fat, 60% from carbohydrates, and 24% from protein). All experiments were approved by the Animal Care Research Committee of the Baylor College of Medicine.

For feces collection, mice were singly housed for 4 days, transferred to fresh cages just before the beginning of dark phase, and fecal pellets produced overnight were collected the next morning before 10:00 AM. Fecal pellets were flash frozen and stored at −80° C. At end of collection the mice were returned to group housing with previous littermates.

Serum samples were collected under fed condition in the morning between 8-10:00 AM. Animals were anaesthetized with isoflurane, blood collected via retro-orbital bleeding, then mice were euthanized by cervical dislocation. Blood samples were centrifuged at 3000 *g* for 15 min at 4° C for serum collection, which were stored at -80° C until metabolome analysis.

### 16S rRNA sequencing analysis

Fecal microbiome analysis was performed as we previous described [65]. Briefly, fecal pellets were homogenized, and microbial DNA was extracted from the homogenate using the PowerSoil DNA extraction kit (MO BIO Laboratories, Carlsbad, CA). The V4 region of 16S rRNA was sequenced on a MiSeq platform (Illumina, San Diego, CA) with a minimum of 800 and an average of 7500 sequences generated per sample. Sequence data were processed as previous described [66]. Taxonomic assignment was performed with RDP as the classifier, and HitDB and SILVA as the selected databases. Bacterial operation taxonomic units (OTUs) were counted for each sample to express the richness of bacterial species with an identity cutoff of 97%. Alpha diversity and beta diversity were calculated using the web-based tool MicrobiomeAnalyst [67]. Comparisons between groups were made at various taxonomic levels.

### Metabolome sample analysis

Untargeted liquid chromatography high resolution accurate mass spectrometry (LC-MS) analysis was performed on a Q Exactive Plus Orbitrap mass spectrometer (Thermo Scientific, Waltham, MA) coupled to a binary pump HPLC (UltiMate 3000, Thermo Scientific) [68]. Full MS spectra were obtained at 70,000 resolution (200 m/z) with a scan range of 50-750 m/z. Full MS followed by ddMS2 scans were obtained at 35,000 resolution (MS1) and 17,500 resolution (MS2) with a 1.5 m/z isolation window and a stepped NCE (20, 40, 60). Samples were maintained at 4° C before injection. The injection volume was 10 μL. Chromatographic separation was achieved on a Synergi Fusion 4μm, 150 mm x 2 mm reverse phase column (Phenomenex, Torrance, CA) maintained at 30° C using a solvent gradient method. Solvent A was water (0.1% formic acid). Solvent B was methanol (0.1% formic acid). The gradient method used was 0-5 min (10% B to 40% B), 5-7 min (40% B to 95% B), 7-9 min (95% B), 9-9.1 min (95% B to 10% B), 9.1-13 min (10% B). The flow rate was 0.4 mL min^-1^. Sample acquisition was performed using Xcalibur (Thermo Scientific, Waltham, Ma, USA). Data analysis was performed with Compound Discoverer 2.1 (Thermo Scientific) and the web-based tool MetaboAnalyst 4.0 [69].

## Supplementary Material

Supplementary Table 1

Supplementary Table 2

## References

[1] Ahima RS. Connecting obesity, aging and diabetes. Nat Med. 2009; 15:996–97. 10.1038/nm0909-996 19734871

[2] Tchkonia T, Morbeck DE, Von Zglinicki T, Van Deursen J, Lustgarten J, Scrable H, Khosla S, Jensen MD, Kirkland JL. Fat tissue, aging, and cellular senescence. Aging Cell. 2010; 9:667–84. 10.1111/j.1474-9726.2010.00608.x 20701600 PMC2941545

[3] Wang JC, Bennett M. Aging and atherosclerosis: mechanisms, functional consequences, and potential therapeutics for cellular senescence. Circ Res. 2012; 111:245–59. 10.1161/CIRCRESAHA.111.261388 22773427

[4] Drew L. An age-old story of dementia. Nature. 2018; 559:S2–S3. 10.1038/d41586-018-05718-5 30046085

[5] Franceschi C, Campisi J. Chronic inflammation (inflammaging) and its potential contribution to age-associated diseases. J Gerontol A Biol Sci Med Sci. 2014 (Suppl 1); 69:S4–9. 10.1093/gerona/glu057 24833586

[6] Devaraj S, Hemarajata P, Versalovic J. The human gut microbiome and body metabolism: implications for obesity and diabetes. Clin Chem. 2013; 59:617–28. 10.1373/clinchem.2012.187617 23401286 PMC3974587

[7] Tremaroli V, Bäckhed F. Functional interactions between the gut microbiota and host metabolism. Nature. 2012; 489:242–49. 10.1038/nature11552 22972297

[8] Thevaranjan N, Puchta A, Schulz C, Naidoo A, Szamosi JC, Verschoor CP, Loukov D, Schenck LP, Jury J, Foley KP, Schertzer JD, Larché MJ, Davidson DJ, et al. Age-Associated Microbial Dysbiosis Promotes Intestinal Permeability, Systemic Inflammation, and Macrophage Dysfunction. Cell Host Microbe. 2017; 21:455–66.e4. 10.1016/j.chom.2017.03.002 28407483 PMC5392495

[9] Biagi E, Nylund L, Candela M, Ostan R, Bucci L, Pini E, Nikkïla J, Monti D, Satokari R, Franceschi C, Brigidi P, De Vos W. Through ageing, and beyond: gut microbiota and inflammatory status in seniors and centenarians. PLoS One. 2010; 5:e10667. 10.1371/journal.pone.0010667 20498852 PMC2871786

[10] Jeffery IB, Lynch DB, O’Toole PW. Composition and temporal stability of the gut microbiota in older persons. ISME J. 2016; 10:170–82. 10.1038/ismej.2015.88 26090993 PMC4681863

[11] O’Toole PW, Jeffery IB. Gut microbiota and aging. Science. 2015; 350:1214–15. 10.1126/science.aac8469 26785481

[12] Kell DB, Oliver SG. The metabolome 18 years on: a concept comes of age. Metabolomics. 2016; 12:148. 10.1007/s11306-016-1108-4 27695392 PMC5009154

[13] Collino S, Montoliu I, Martin FP, Scherer M, Mari D, Salvioli S, Bucci L, Ostan R, Monti D, Biagi E, Brigidi P, Franceschi C, Rezzi S. Metabolic signatures of extreme longevity in northern Italian centenarians reveal a complex remodeling of lipids, amino acids, and gut microbiota metabolism. PLoS One. 2013; 8:e56564. 10.1371/journal.pone.0056564 23483888 PMC3590212

[14] Tomás-Loba A, Bernardes de Jesus B, Mato JM, Blasco MA. A metabolic signature predicts biological age in mice. Aging Cell. 2013; 12:93–101. 10.1111/acel.12025 23107558 PMC3552107

[15] Houtkooper RH, Argmann C, Houten SM, Cantó C, Jeninga EH, Andreux PA, Thomas C, Doenlen R, Schoonjans K, Auwerx J. The metabolic footprint of aging in mice. Sci Rep. 2011; 1:134. 10.1038/srep00134 22355651 PMC3216615

[16] Viltard M, Durand S, Pérez-Lanzón M, Aprahamian F, Lefevre D, Leroy C, Madeo F, Kroemer G, Friedlander G. The metabolomic signature of extreme longevity: naked mole rats versus mice. Aging (Albany NY). 2019; 11:4783–800. 10.18632/aging.102116 31346149 PMC6682510

[17] Cheng S, Larson MG, McCabe EL, Murabito JM, Rhee EP, Ho JE, Jacques PF, Ghorbani A, Magnusson M, Souza AL, Deik AA, Pierce KA, Bullock K, et al. Distinct metabolomic signatures are associated with longevity in humans. Nat Commun. 2015; 6:6791. 10.1038/ncomms7791 25864806 PMC4396657

[18] Srivastava S. Emerging insights into the metabolic alterations in aging using metabolomics. Metabolites. 2019; 9:301. 10.3390/metabo9120301 31847272 PMC6950098

[19] Wikoff WR, Anfora AT, Liu J, Schultz PG, Lesley SA, Peters EC, Siuzdak G. Metabolomics analysis reveals large effects of gut microflora on mammalian blood metabolites. Proc Natl Acad Sci USA. 2009; 106:3698–703. 10.1073/pnas.0812874106 19234110 PMC2656143

[20] Kim BR, Shin J, Guevarra R, Lee JH, Kim DW, Seol KH, Lee JH, Kim HB, Isaacson R. Deciphering diversity indices for a better understanding of microbial communities. J Microbiol Biotechnol. 2017; 27:2089–93. 10.4014/jmb.1709.09027 29032640

[21] Brunt VE, Gioscia-Ryan RA, Richey JJ, Zigler MC, Cuevas LM, Gonzalez A, Vázquez-Baeza Y, Battson ML, Smithson AT, Gilley AD, Ackermann G, Neilson AP, Weir T, et al. Suppression of the gut microbiome ameliorates age-related arterial dysfunction and oxidative stress in mice. J Physiol. 2019; 597:2361–78. 10.1113/JP277336 30714619 PMC6487935

[22] Scott KA, Ida M, Peterson VL, Prenderville JA, Moloney GM, Izumo T, Murphy K, Murphy A, Ross RP, Stanton C, Dinan TG, Cryan JF. Revisiting metchnikoff: age-related alterations in microbiota-gut-brain axis in the mouse. Brain Behav Immun. 2017; 65:20–32. 10.1016/j.bbi.2017.02.004 28179108

[23] Sovran B, Hugenholtz F, Elderman M, Van Beek AA, Graversen K, Huijskes M, Boekschoten MV, Savelkoul HF, De Vos P, Dekker J, Wells JM. Age-associated impairment of the mucus barrier function is associated with profound changes in microbiota and immunity. Sci Rep. 2019; 9:1437. 10.1038/s41598-018-35228-3 30723224 PMC6363726

[24] Hoffman JD, Parikh I, Green SJ, Chlipala G, Mohney RP, Keaton M, Bauer B, Hartz AM, Lin AL. Age drives distortion of brain metabolic, vascular and cognitive functions, and the gut microbiome. Front Aging Neurosci. 2017; 9:298. 10.3389/fnagi.2017.00298 28993728 PMC5622159

[25] Claesson MJ, Cusack S, O’Sullivan O, Greene-Diniz R, de Weerd H, Flannery E, Marchesi JR, Falush D, Dinan T, Fitzgerald G, Stanton C, van Sinderen D, O’Connor M, et al. Composition, variability, and temporal stability of the intestinal microbiota of the elderly. Proc Natl Acad Sci USA. 2011 (Suppl 1); 108:4586–91. 10.1073/pnas.1000097107 20571116 PMC3063589

[26] Claesson MJ, Jeffery IB, Conde S, Power SE, O’Connor EM, Cusack S, Harris HM, Coakley M, Lakshminarayanan B, O’Sullivan O, Fitzgerald GF, Deane J, O’Connor M, et al. Gut microbiota composition correlates with diet and health in the elderly. Nature. 2012; 488:178–84. 10.1038/nature11319 22797518

[27] Kelly TN, Bazzano LA, Ajami NJ, He H, Zhao J, Petrosino JF, Correa A, He J. Gut microbiome associates with lifetime cardiovascular disease risk profile among bogalusa heart study participants. Circ Res. 2016; 119:956–64. 10.1161/CIRCRESAHA.116.309219 27507222 PMC5045790

[28] Mariat D, Firmesse O, Levenez F, Guimarăes V, Sokol H, Doré J, Corthier G, Furet JP. The firmicutes/bacteroidetes ratio of the human microbiota changes with age. BMC Microbiol. 2009; 9:123. 10.1186/1471-2180-9-123 19508720 PMC2702274

[29] Ley RE, Turnbaugh PJ, Klein S, Gordon JI. Microbial ecology: human gut microbes associated with obesity. Nature. 2006; 444:1022–23. 10.1038/4441022a 17183309

[30] Verdam FJ, Fuentes S, de Jonge C, Zoetendal EG, Erbil R, Greve JW, Buurman WA, de Vos WM, Rensen SS. Human intestinal microbiota composition is associated with local and systemic inflammation in obesity. Obesity (Silver Spring). 2013; 21:E607–15. 10.1002/oby.20466 23526699

[31] Jakobsson HE, Rodríguez-Piñeiro AM, Schütte A, Ermund A, Boysen P, Bemark M, Sommer F, Bäckhed F, Hansson GC, Johansson ME. The composition of the gut microbiota shapes the colon mucus barrier. EMBO Rep. 2015; 16:164–77. 10.15252/embr.201439263 25525071 PMC4328744

[32] Fransen F, van Beek AA, Borghuis T, Aidy SE, Hugenholtz F, van der Gaast-de Jongh C, Savelkoul HF, De Jonge MI, Boekschoten MV, Smidt H, Faas MM, de Vos P. Aged gut microbiota contributes to systemical inflammaging after transfer to germ-free mice. Front Immunol. 2017; 8:1385. 10.3389/fimmu.2017.01385 29163474 PMC5674680

[33] Derrien M, Belzer C, de Vos WM. Akkermansia muciniphila and its role in regulating host functions. Microb Pathog. 2017; 106:171–81. 10.1016/j.micpath.2016.02.005 26875998

[34] Shin NR, Lee JC, Lee HY, Kim MS, Whon TW, Lee MS, Bae JW. An increase in the akkermansia spp. Population induced by metformin treatment improves glucose homeostasis in diet-induced obese mice. Gut. 2014; 63:727–35. 10.1136/gutjnl-2012-303839 23804561

[35] Kang CS, Ban M, Choi EJ, Moon HG, Jeon JS, Kim DK, Park SK, Jeon SG, Roh TY, Myung SJ, Gho YS, Kim JG, Kim YK. Extracellular vesicles derived from gut microbiota, especially akkermansia muciniphila, protect the progression of dextran sulfate sodium-induced colitis. PLoS One. 2013; 8:e76520. 10.1371/journal.pone.0076520 24204633 PMC3811976

[36] Ganesh BP, Klopfleisch R, Loh G, Blaut M. Commensal akkermansia muciniphila exacerbates gut inflammation in salmonella typhimurium-infected gnotobiotic mice. PLoS One. 2013; 8:e74963. 10.1371/journal.pone.0074963 24040367 PMC3769299

[37] McReynolds MR, Chellappa K, Baur JA. Age-related NAD^+^ decline. Exp Gerontol. 2020; 134:110888. 10.1016/j.exger.2020.110888 32097708 PMC7442590

[38] Mouchiroud L, Houtkooper RH, Auwerx J. NAD⁺ metabolism: a therapeutic target for age-related metabolic disease. Crit Rev Biochem Mol Biol. 2013; 48:397–408. 10.3109/10409238.2013.789479 23742622 PMC3858599

[39] Yoshino J, Mills KF, Yoon MJ, Imai S. Nicotinamide mononucleotide, a key NAD(+) intermediate, treats the pathophysiology of diet- and age-induced diabetes in mice. Cell Metab. 2011; 14:528–36. 10.1016/j.cmet.2011.08.014 21982712 PMC3204926

[40] Gomes AP, Price NL, Ling AJ, Moslehi JJ, Montgomery MK, Rajman L, White JP, Teodoro JS, Wrann CD, Hubbard BP, Mercken EM, Palmeira CM, de Cabo R, et al. Declining NAD(+) induces a pseudohypoxic state disrupting nuclear-mitochondrial communication during aging. Cell. 2013; 155:1624–38. 10.1016/j.cell.2013.11.037 24360282 PMC4076149

[41] Massudi H, Grant R, Braidy N, Guest J, Farnsworth B, Guillemin GJ. Age-associated changes in oxidative stress and NAD+ metabolism in human tissue. PLoS One. 2012; 7:e42357. 10.1371/journal.pone.0042357 22848760 PMC3407129

[42] Bogan KL, Brenner C. Nicotinic acid, nicotinamide, and nicotinamide riboside: a molecular evaluation of NAD+ precursor vitamins in human nutrition. Annu Rev Nutr. 2008; 28:115–30. 10.1146/annurev.nutr.28.061807.155443 18429699

[43] Martens CR, Denman BA, Mazzo MR, Armstrong ML, Reisdorph N, McQueen MB, Chonchol M, Seals DR. Chronic nicotinamide riboside supplementation is well-tolerated and elevates NAD^+^ in healthy middle-aged and older adults. Nat Commun. 2018; 9:1286. 10.1038/s41467-018-03421-7 29599478 PMC5876407

[44] de Picciotto NE, Gano LB, Johnson LC, Martens CR, Sindler AL, Mills KF, Imai S, Seals DR. Nicotinamide mononucleotide supplementation reverses vascular dysfunction and oxidative stress with aging in mice. Aging Cell. 2016; 15:522–30. 10.1111/acel.12461 26970090 PMC4854911

[45] Elhassan YS, Kluckova K, Fletcher RS, Schmidt MS, Garten A, Doig CL, Cartwright DM, Oakey L, Burley CV, Jenkinson N, Wilson M, Lucas SJ, Akerman I, et al. Nicotinamide riboside augments the aged human skeletal muscle NAD^+^ metabolome and induces transcriptomic and anti-inflammatory signatures. Cell Rep. 2019; 28:1717–28.e6. 10.1016/j.celrep.2019.07.043 31412242 PMC6702140

[46] Tasca CI, Lanznaster D, Oliveira KA, Fernández-Dueñas V, Ciruela F. Neuromodulatory effects of guanine-based purines in health and disease. Front Cell Neurosci. 2018; 12:376. 10.3389/fncel.2018.00376 30459558 PMC6232889

[47] Ivanisevic J, Stauch KL, Petrascheck M, Benton HP, Epstein AA, Fang M, Gorantla S, Tran M, Hoang L, Kurczy ME, Boska MD, Gendelman HE, Fox HS, Siuzdak G. Metabolic drift in the aging brain. Aging (Albany NY). 2016; 8:1000–20. 10.18632/aging.100961 27182841 PMC4931850

[48] Le Floc’h N, Otten W, Merlot E. Tryptophan metabolism, from nutrition to potential therapeutic applications. Amino Acids. 2011; 41:1195–205. 10.1007/s00726-010-0752-7 20872026

[49] Roager HM, Licht TR. Microbial tryptophan catabolites in health and disease. Nat Commun. 2018; 9:3294. 10.1038/s41467-018-05470-4 30120222 PMC6098093

[50] Yao K, Fang J, Yin YL, Feng ZM, Tang ZR, Wu G. Tryptophan metabolism in animals: important roles in nutrition and health. Front Biosci (Schol Ed). 2011; 3:286–97. 10.2741/s152 21196377

[51] Keszthelyi D, Troost FJ, Masclee AA. Understanding the role of tryptophan and serotonin metabolism in gastrointestinal function. Neurogastroenterol Motil. 2009; 21:1239–49. 10.1111/j.1365-2982.2009.01370.x 19650771

[52] Gostner JM, Geisler S, Stonig M, Mair L, Sperner-Unterweger B, Fuchs D. Tryptophan metabolism and related pathways in psychoneuroimmunology: the impact of nutrition and lifestyle. Neuropsychobiology. 2020; 79:89–99. 10.1159/000496293 30808841

[53] Bansal T, Englert D, Lee J, Hegde M, Wood TK, Jayaraman A. Differential effects of epinephrine, norepinephrine, and indole on escherichia coli O157:H7 chemotaxis, colonization, and gene expression. Infect Immun. 2007; 75:4597–607. 10.1128/IAI.00630-07 17591798 PMC1951185

[54] Kohli N, Crisp Z, Riordan R, Li M, Alaniz RC, Jayaraman A. The microbiota metabolite indole inhibits salmonella virulence: involvement of the PhoPQ two-component system. PLoS One. 2018; 13:e0190613. 10.1371/journal.pone.0190613 29342189 PMC5771565

[55] Bommarius B, Anyanful A, Izrayelit Y, Bhatt S, Cartwright E, Wang W, Swimm AI, Benian GM, Schroeder FC, Kalman D. A family of indoles regulate virulence and Shiga toxin production in pathogenic E. Coli. PLoS One. 2013; 8:e54456. 10.1371/journal.pone.0054456 23372726 PMC3553163

[56] Zelante T, Iannitti RG, Cunha C, De Luca A, Giovannini G, Pieraccini G, Zecchi R, D’Angelo C, Massi-Benedetti C, Fallarino F, Carvalho A, Puccetti P, Romani L. Tryptophan catabolites from microbiota engage aryl hydrocarbon receptor and balance mucosal reactivity via interleukin-22. Immunity. 2013; 39:372–85. 10.1016/j.immuni.2013.08.003 23973224

[57] Shimada Y, Kinoshita M, Harada K, Mizutani M, Masahata K, Kayama H, Takeda K. Commensal bacteria-dependent indole production enhances epithelial barrier function in the colon. PLoS One. 2013; 8:e80604. 10.1371/journal.pone.0080604 24278294 PMC3835565

[58] Safe S, Jayaraman A, Chapkin RS. Ah receptor ligands and their impacts on gut resilience: structure-activity effects. Crit Rev Toxicol. 2020; 50:463–73. 10.1080/10408444.2020.1773759 32597352 PMC7773274

[59] Gao J, Xu K, Liu H, Liu G, Bai M, Peng C, Li T, Yin Y. Impact of the gut microbiota on intestinal immunity mediated by tryptophan metabolism. Front Cell Infect Microbiol. 2018; 8:13. 10.3389/fcimb.2018.00013 29468141 PMC5808205

[60] Sonowal R, Swimm A, Sahoo A, Luo L, Matsunaga Y, Wu Z, Bhingarde JA, Ejzak EA, Ranawade A, Qadota H, Powell DN, Capaldo CT, Flacker JM, et al. Indoles from commensal bacteria extend healthspan. Proc Natl Acad Sci USA. 2017; 114:E7506–15. 10.1073/pnas.1706464114 28827345 PMC5594673

[61] Bansal T, Alaniz RC, Wood TK, Jayaraman A. The bacterial signal indole increases epithelial-cell tight-junction resistance and attenuates indicators of inflammation. Proc Natl Acad Sci USA. 2010; 107:228–33. 10.1073/pnas.0906112107 19966295 PMC2806735

[62] Gagliano N, Arosio B, Santambrogio D, Balestrieri MR, Padoani G, Tagliabue J, Masson S, Vergani C, Annoni G. Age-dependent expression of fibrosis-related genes and collagen deposition in rat kidney cortex. J Gerontol A Biol Sci Med Sci. 2000; 55:B365–72. 10.1093/gerona/55.8.b365 10952357

[63] Wu G, Bazer FW, Burghardt RC, Johnson GA, Kim SW, Knabe DA, Li P, Li X, McKnight JR, Satterfield MC, Spencer TE. Proline and hydroxyproline metabolism: implications for animal and human nutrition. Amino Acids. 2011; 40:1053–63. 10.1007/s00726-010-0715-z 20697752 PMC3773366

[64] He B, Nohara K, Ajami NJ, Michalek RD, Tian X, Wong M, Losee-Olson SH, Petrosino JF, Yoo SH, Shimomura K, Chen Z. Transmissible microbial and metabolomic remodeling by soluble dietary fiber improves metabolic homeostasis. Sci Rep. 2015; 5:10604. 10.1038/srep10604 26040234 PMC4455235

[65] Wu CS, Wei Q, Wang H, Kim DM, Balderas M, Wu G, Lawler J, Safe S, Guo S, Devaraj S, Chen Z, Sun Y. Protective effects of ghrelin on fasting-induced muscle atrophy in aging mice. J Gerontol A Biol Sci Med Sci. 2020; 75:621–30. 10.1093/gerona/gly256 30407483 PMC7328200

[66] Pahwa R, Balderas M, Jialal I, Chen X, Luna RA, Devaraj S. Gut microbiome and inflammation: a study of diabetic inflammasome-knockout mice. J Diabetes Res. 2017; 2017:6519785. 10.1155/2017/6519785 29435463 PMC5804379

[67] Chong J, Liu P, Zhou G, Xia J. Using MicrobiomeAnalyst for comprehensive statistical, functional, and meta-analysis of microbiome data. Nat Protoc. 2020; 15:799–821. 10.1038/s41596-019-0264-1 31942082

[68] Weindel CG, Bell SL, Vail KJ, West KO, Patrick KL, Watson RO. LRRK2 maintains mitochondrial homeostasis and regulates innate immune responses to Mycobacterium tuberculosis. Elife. 2020; 9:e51071. 10.7554/eLife.51071 32057291 PMC7159881

[69] Chong J, Wishart DS, Xia J. Using MetaboAnalyst 4.0 for comprehensive and integrative metabolomics data analysis. Curr Protoc Bioinformatics. 2019; 68:e86. 10.1002/cpbi.86 31756036

